# High frequency of transient congenital hypothyroidism among infants referred for suspected congenital hypothyroidism from the Turkish National screening program: thyroxine dose may guide the prediction of transients

**DOI:** 10.1007/s40618-024-02348-9

**Published:** 2024-03-28

**Authors:** Y. Özer, A. Anık, U. Sayılı, U. Tercan, R. Deveci Sevim, S. Güneş, M. Buhur Pirimoğlu, S. Elmaoğulları, İ. Dündar, D. Ökdemir, Ö. Besci, A. Jalilova, D. Çiçek, B. Singin, Ş. E. Ulu, H. Turan, S. Albayrak, Z. Kocabey Sütçü, B. S. Eklioğlu, E. Eren, S. Çetinkaya, Ş. Savaş-Erdeve, İ. Esen, K. Demir, Ş. Darcan, N. Hatipoğlu, M. Parlak, F. Dursun, Z. Şıklar, M. Berberoğlu, M. Keskin, Z. Orbak, B. Tezel, E. Yürüker, B. Keskinkılıç, F. Kara, E. Erginöz, F. Darendeliler, O. Evliyaoğlu

**Affiliations:** 1 Department of Pediatric Endocrinology, Cerrahpasa Faculty of Medicine, Istanbul University-Cerrahpasa, Istanbul, Turkey; 2 Department of Pediatric Endocrinology, Faculty of Medicine, Aydın Adnan Menderes University, Aydın, Turkey; 3 Department of Public Health, Cerrahpasa Faculty of Medicine, Istanbul University-Cerrahpasa, Istanbul, Turkey; 4 Department of Pediatric Endocrinology, Istanbul Medical Faculty, Istanbul University, Istanbul, Turkey; 5 Department of Pediatric Endocrinology, Faculty of Medicine, Bursa Uludağ University, Bursa, Turkey; 6 Department of Pediatric Endocrinology, University of Health Sciences Turkey, Dr. Sami Ulus Maternity and Children’s Research and Training Hospital, Ankara, Turkey; 7 Department of Pediatric Endocrinology, Malatya Education and Research Hospital, Malatya, Turkey; 8 Department of Pediatric Endocrinology, Faculty of Medicine, Fırat University, Elazığ, Turkey; 9 Department of Pediatric Endocrinology, Faculty of Medicine, Dokuz Eylül University, İzmir, Turkey; 10 Department of Pediatric Endocrinology, Faculty of Medicine, Ege University, Izmir, Turkey; 11 Department of Pediatric Endocrinology, Faculty of Medicine, Erciyes University, Kayseri, Turkey; 12 Department of Pediatric Endocrinology, Faculty of Medicine, Akdeniz University, Antalya, Turkey; 13 Departments of Pediatric Endocrinology, Ümraniye Training and Research Hospital, Istanbul, Turkey; 14 Department of Pediatric Endocrinology, Faculty of Medicine, Gaziantep University, Gaziantep, Turkey; 15 Department of Pediatric Endocrinology, Basaksehir Cam and Sakura City Hospital, Istanbul, Turkey; 16 Department of Pediatric Endocrinology, Meram Faculty of Medicine, Necmettin Erbakan University, Konya, Turkey; 17 Department of Pediatric Endocrinology, Faculty of Medicine, Ankara University, Ankara, Turkey; 18 Department of Pediatric Endocrionology, Faculty of Medicine, Atatürk University, Erzurum, Turkey; 19 Department of Child and Adolescents Health, Ministry of Health, General Directorate of Public Health, Ankara, Turkey

**Keywords:** Neonatal screening, Transient congenital hypothyroidism, Permanent congenital hypothyroidism, Frequency, Etiology, Thyroxine

## Abstract

**Purpose:**

We aimed to determine the frequency of transient congenital hypothyroidism (TCH) in 17 participating centers in Türkiye, evaluate the etiological distribution in permanent congenital hypothyroidism (PCH) cases, and investigate the role of laboratory and clinical findings in predicting TCH.

**Methods:**

This retrospective observational multicenter study included patients from 17 pediatric endocrinology centers identified by “National Newborn Screening Program” (NNSP) who were born in 2015 and followed for 6 years. Demographic, clinical, and laboratory information of the cases were compiled through the database http://cedd.saglik-network.org (CEDD-NET).

**Results:**

Of the 239 cases initially treated for CH, 128 (53.6%) were determined as transient in whom a trial of levothyroxine (LT4) withdrawal was performed at a median age of 36 (34–38) months. Among the patients with PCH (*n* = 111), thyroid dysgenesis was diagnosed in 39.6% (*n* = 44). The predictive factors for TCH were: LT4 dose at the withdrawal of treatment, and initial newborn blood screening (NBS)-TSH level. Based on the receiver operating characteristic (ROC) curve analysis to predict optimal cut-offs for TCH predictors, LT4 dose < 2.0 µg/kg/day at treatment discontinuation was predictive for TCH and was associated with 94.5% specificity and 55.7% sensitivity, with an area under the curve (AUC) of 0.802. The initial NBS-TSH level value < 45 µIU/mL was predictive for TCH with 93.1% specificity and 45.5% sensitivity, with an AUC of 0.641. In patients with eutopic thyroid gland only LT4 dose < 1.1 µg/kg/day at withdrawal time was predictive for TCH with 84.7% sensitivity and 40.4% specificity, with an AUC of 0.750.

**Conclusion:**

According to our national follow-up data, the frequency of TCH was 53.6%. We determined the LT4 dose < 2.0 µg/kg/day at discontinuation of treatment and the initial NBS-TSH level < 45 µIU/mL as the best cut-off limits to predict TCH.

## Introduction

Congenital hypothyroidism (CH) is the most common endocrine disorder in children. Newborn blood screening (NBS) programs have improved neurodevelopmental outcomes with earlier diagnosis and treatment of CH. In iodine-replete populations, primary CH is usually due to thyroid dysgenesis (80%) and rarely to dyshormonogenesis [1]. Although the incidence of CH varies regionally, it is reported to be around 1:2000 in western populations [2, 3]. The probable incidence of CH was reported as 1/650 between 2008 and 2010 in Türkiye [4]. Although transient forms were not excluded in different regions of Türkiye, the incidence of CH was reported as 1/2326 in 2005 [5] and 1/2183 in 2011 [6].

Congenital hypothyroidism is classified as permanent CH (PCH) or transient CH (TCH), depending on whether treatment is continually needed [7]. With the increase in the incidence of CH as a result of the widespread use of NBS, the incidence of TCH is also increasing. The increased incidence is explained by the strategy changes in NBS with the introduction of lower thresholds to increase sensitivity and increased preterm deliveries with increased survival rates of infants born preterm [2, 8–10]. In some studies, more than half of the cases with CH with eutopic thyroid gland have been reported as transient, although iodine status has not been reported in these studies [7, 11–14]. Transient CH can be caused by genetic (*DUOX2* and *DUOXA2* mutations) and environmental factors (maternal or infant iodine deficiency and excess, TSH receptor blocking antibodies acquired through transplacental transmission, maternal antithyroid drugs) [1, 15].

The etiology of CH is important in determining the prognosis and clinical management. If there is no etiologic diagnosis (especially in patients with gland in situ and presumed isolated central CH) re-evaluation of the hypothalamo–pituitary–thyroid axis is recommended in patients at the age of 3 years. For precise diagnosis, levothyroxine (LT4) treatment is phased out. Four weeks later, thyroid functions are re-evaluated and if serum TSH is lower than 10 mU/L then TCH is confirmed [16–18]. If thyroid functions are normal despite low LT4 doses, re-evaluation can be performed before 2 years of age, thus in the recent European Society for Pediatric Endocrinology (ESPE) and the European Society for Endocrinology guidelines re-evaluation is suggested at 6 months in patients with no PCH diagnosis and an in situ gland who needs an LT4 dose of < 3 μg/kg/day [17]. However, the latest American Pediatric Academy guidelines suggest LT4 withdrawal trial after 3 years of age [18]. To avoid unnecessary treatment, the focus has been on the search for markers to reliably differentiate PCH and TCH [12, 13, 19–22]. Findings at the time of diagnosis alone may not be sufficient to predict TCH. Several studies have been conducted to identify predictors of TCH [7, 12, 13, 22–24]. However, there is no consensus on TCH predictors.

Unfortunately, there are no nationwide data regarding the frequency of TCH in Türkiye. Our study aimed to determine the frequency of TCH, etiological distribution of PCH, and predictive markers of TCH in CH cases who were detected in the National Neonatal Screening Program (NNSP) for CH, only involving 17 participating centers in Türkiye.

## Materials and methods

Seventeen pediatric endocrinology centers participated in the study between January 2021, and January 2022. In this retrospective study, medical records of cases born between Jan 01, 2015, and Dec 31, 2015, who were diagnosed as CH by referral from NNSP to pediatric endocrinology centers were involved. All patients were followed for 6 years. Demographic, clinical, genetic, and laboratory information of the cases was compiled through a CEDD-NET Data System (http://cedd.saglik-network.org/).

The inclusion criteria were as follows: cases referred to pediatric endocrinology centers with the suspicion of CH by NNSP. The exclusion criteria were as follows: insufficient data to diagnose CH, inadequate follow-up, and patients with genetic syndromes/chromosomal abnormalities.

Data on demographic and perinatal information (gender, gestational age, birth weight, consanguinity between parents, and admission to the neonatal intensive care unit), and family history of CH or thyroid disease, were obtained through a review of medical records. Age, thyroid function tests, and LT4 dose at diagnosis and during the LT4 withdrawal trial were recorded.

The cases were categorized as CH, isolated TSH elevation, and normal according to their serum thyrotropin (TSH) and free T4 (FT4) levels at the initial evaluations. Cases diagnosed with CH were treated with LT4 until the age of 3 years or before and were re-evaluated after 4 weeks of LT4 discontinuation. The 6 month follow-up of the patients who underwent the LT4 withdrawal trial was recorded. It was categorized as PCH or TCH after 6 months of follow-up. Permanent CH was diagnosed in cases where treatment was restarted due to high TSH (with TSH > 8 µIU/mL) after a 4 weeks cessation trial of LT4 treatment and in cases with a definitive diagnosis of CH (dysgenesis detected in thyroid ultrasonography or drug dose increased in follow-up). Presence of normal thyroid function tests at 1, 3, and 6 months after discontinuation of LT4 treatment was considered as TCH.

Newborn screening was performed by measuring TSH with Trimaris neonatal TSH FEIA kits using filter paper blood in a dried bloodstain sample taken 3–5 days after birth. A fluorescent enzyme immunoassay based on the sandwich principle of two monoclonal antibodies specific for TSH was used. The sensitivity of the TSH test was 0.5–1.1 µIU/mL. Newborn screening results were provided by the Turkish Public Health Directorate. The TSH cut-off value in NNSP is 5.5 µIU/mL. According to the NNSP, a second control heel pierce is performed for values between 5.5 and 20 µIU/mL. All cases with a TSH concentration higher than 20 µIU/mL in the first sample or higher than 5.5 µIU/mL in repeated blood samples are referred to the pediatric endocrinology center for evaluation. For confirmatory testing, TSH and FT4 levels were measured in peripheral venous blood samples at local laboratories. Since it was studied in different laboratories, TSH values above 100 µIU/mL were accepted as 100 µIU/mL. The diagnosis of CH and the treatment decision were in accordance with the guidelines set by the ESPE [16].

Thyroid ultrasonography or Technetium-99 (Tc-99 m) thyroid scan was performed by the local center at the time of diagnosis or follow-up. The volume of the thyroid lobes were calculated using the formula 0.479 × length (cm) × width (cm) × thickness (cm). Total thyroid volume was the sum of the volumes of the two lobes and compared with reference values in the literature [25]. Cases with normal thyroid gland size and location on thyroid ultrasonography and/or thyroid scintigraphy were defined as eutopic CH. Cases with complete agenesis, hemiagenesis, gland hypoplasia, or ectopic thyroid were defined as dysgenesis. Patients with a thyroid gland that did not show dysgenesis in ultrasound studies were diagnosed as “possible dyshormonogenesis”.

An iodine level < 100 µg/L was considered evidence of iodine deficiency. An iodine level above 200 µg/L was considered evidence of iodine excess.

This study complied with the recommendations of the Declaration of Helsinki and was approved by the Cerrahpaşa Medical Faculty Ethics Committee (Project Number: 4th Mar 2020; 37494).

## Statistical analyses

The Statistical Package for the Social Science version 21.0 for Windows (IBM Corp., Armonk, NY, USA) was used for data evaluation and analysis. Categorical variables are presented as frequencies (*n*) and percentages (%), and numeric variables are presented as median (25–75p) values. The Kolmogorov–Smirnov test was used to evaluate normality. The Chi-square test or Fisher’s exact test was used to compare the categorical variables. The Mann–Whitney *U* test was used to compare continuous variables between two independent samples. Multivariate logistic regression was performed to determine the risk ratio for PCH. The Hosmer–Lemeshow test was used to evaluate the model fit. A receiver operating characteristic (ROC) analysis is used to evaluate area under the curve (AUC), cut-off value, sensitivity, and specificity of laboratory parameters on CH. The significance level of statistical tests was set at *p* < 0.05.

## Results

**Fig. 1:**
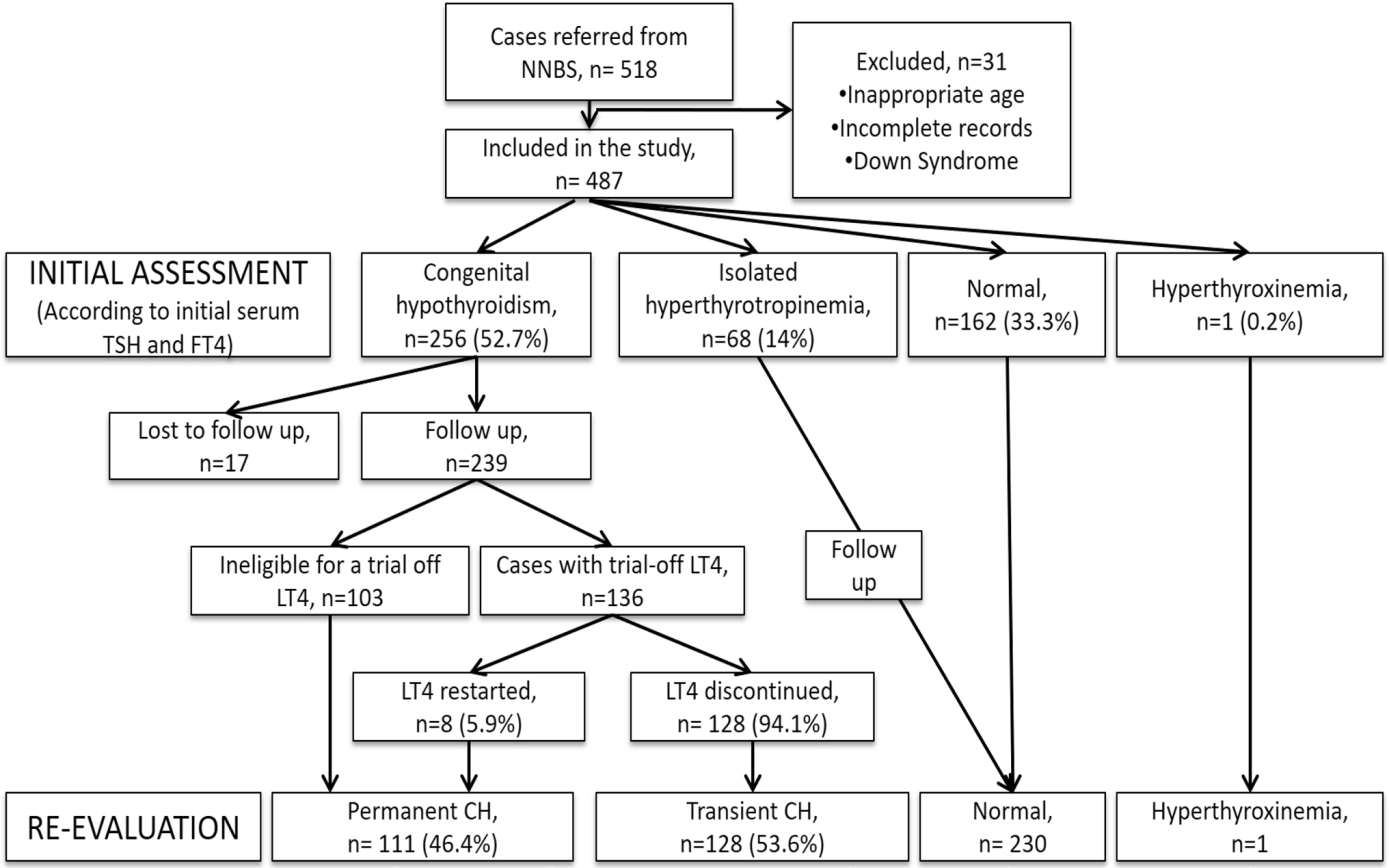
Flow diagram cases with permanent congenital hypothyroidism (PCH) and transient congenital hypothyroidism (TCH)

In 2015, 1,292,703 newborns were screened in the national screening program. 13,556 newborns were referred for serum TSH and FT4 evaluation, and 3606 newborns were put in follow-up with suspected CH. However, the precise incidence of CH in Türkiye is not known. Although we do not have the data on the whole screening program, we have the data on newborns identified with suspected CH and referred to 1 of 17 pediatric endocrinology centers. This study gives the frequency of TCH in the studied group. After excluding 31 patients due to insufficient data or trisomy 21, 487 children coming from the NNSP were involved in the study. Confirmatory thyroid function tests of 162 (33.3%) cases were normal. Sixty-eight (14%) cases had transient hyperthyrotropinemia, which resolved in a median of 35 (21–49.5) days without any treatment. Finally, 256 (121 male, 118 female) cases were diagnosed as CH and LT4 treatment was started. In the follow-up, an LT4 cessation trial was performed in 136 of 239 patients (Fig. 1). The other 103 patients were deemed ineligible for LT4 withdrawal based on dysgenesis detected in thyroid ultrasonography or LT4 dose increased in the follow-up period. The median age at LT4 withdrawal was 36 (34–38) months.

In TCH cases, LT4 treatment withdrawal trials were performed after 3 years of age in 83 (64.9%), between 2 and 3 years of age in 29 (22.7%), and before the age of 2 years in 16 (12.5%) of the cases. Trial off failed in 8 patients and LT4 was restarted. The LT4 cessation attempt was successful in 94.1% of cases. The diagnostic flow diagram of cases with PCH and TCH is shown in Fig. 1.

Permanent CH was diagnosed in patients who could not undergo LT4 withdrawal (*n* = 103) and who had been restarted LT4 (*n* = 8) because of elevated TSH after withdrawal; a total of 111 cases (46.4%) were diagnosed with PCH. After the LT4 withdrawal trial, 128 cases (53.6%) with TSH < 10 µIU/mL for 6 months were diagnosed as TCH (Fig. 1). Thyroid ultrasonography was performed in all infants whose treatment was initiated (*n* = 239) at the time of diagnosis. Total thyroid volume was lower in the PCH group than in the TCH group (*p* < 0.001). According to sonographic findings, 39.6% (*n* = 44) of the cases with PCH were diagnosed as dysgenesis (18 hypoplasia, 16 ageneses, 5 hemiagenesis, 5 ectopia). The remaining 67 (60.4%) cases with eutopic thyroid gland were classified as possible dyshormonogenesis and it was the most common etiology in PCH. While hemiagenesis was detected in one patient with TCH, the others had eutopic thyroid glands (Table 2). In patients with thyroid dysgenesis gender distribution was 1.9 (female:male), and in patients with presumed dyshormonogenesis gender distribution was 0.8 (female:male).

Thyroid scintigraphy and/or perchlorate discharge test were performed in 17 cases with CH and 4 cases were diagnosed as agenesis/hemiagenesis, 4 cases with ectopia, 2 cases with dyshormonogenesis.

Genetic analysis was performed in five cases. *TG* and *SLC6A7* variants were detected in one case each in the permanent group and the *DUOX2* variant in one case in the transient group.

Among the patients with eutopic thyroid gland 67 (34.5%) were diagnosed as PCH, and 127 (65.5%) were diagnosed as TCH group. Among the patients with thyroid dysgenesis, only one was diagnosed as TCH, who had hemiagenesis.

Among the 239 cases, there was a history of prematurity in 16.7% (*n* = 40) and congenital malformations in 3.7% (*n* = 9), in which cardiovascular and urinary defects were found to be predominant. In the cases with PCH and TCH, there was a history of prematurity in 14.4% (*n* = 16) and 18.8% (*n* = 24), respectively. Congenital malformations were identified in 3.6% (*n* = 4) of the patients with thyroid dysgenesis and in 3.9% (*n* = 5) of those with presumed thyroid dyshormonogenesis. Parental consanguinity was found in 25.4% of PCH cases and in 21.3% of TCH cases (*p* = 0.559). There was no statistically significant difference between the PCH and TCH groups in terms of gender, birth weight, gestational age, prematurity, hypoxia, age at diagnosis, and age at onset of treatment. Maternal history of thyroid disease was not different between the PCH and TCH groups (*p* = 0.054). Fifteen (13.5%) cases with PCH had a maternal history of thyroid disease (14 hypothyroidism, 1 papillary thyroid carcinoma). Seven patients (5.5%) with TCH had a maternal history of thyroid disease (6 hypothyroidism, 1 Grave’s disease). Maternal history of thyroid disease was significantly higher in the eutopic PCH group compared to the eutopic TCH group (*p* = 0.014). The demographic features of cases with PCH and TCH are shown in Table 1.

**Table 1:** Demographic features of the cases with PCH and TCH

	PCH (*n* = 111)	TCH (*n* = 128)	*p*-value	Eutopic PCH (*n* = 67)	Eutopic TCH (*n* = 127)	*p*-value
Current age (months)*	76 (72–80)	75 (70–79)	0.112^&^	76 (72–79)	75 (70–79)	0.391^&^
Female/male, *n* (%)	58 (52.3)/53 (47.7)	60 (46.9)/68 (53.1)	0.407^#^	29 (43.3)/38 (56.7)	59 (46.5)/68 (53.5)	0.572^#^
Parental consanguinity, *n* (%)	16/63 (25.4)	17/80 (21.3)	0.559^#^	12/39 (30.8)	16/78 (20.5)	0.220^#^
Perinatal history						
Birth weight (grams)*	3150 (2850–3510)	3160 (2840–3500)	0.825^&^	3235 (2800–3500)	3150 (2800–3500)	0.816^&^
Gestational age (weeks)*	39(38–40)	39 (38–40)	0.948^&^	39 (38–40)	39 (38–40)	0.887^&^
Prematurity, *n* (%)	16 (14.4)	24 (18.8)	0.444^#^	9 (13.4)	24 (18.9)	0.395^#^
Hypoxia, *n* (%)	3 (2.7)	5 (3.9)	0.724 ^¶^	1 (1.5)	5 (3.9)	0.665^¶^
Admission to NICU, *n* (%)	14 (12.6)	14 (10.9)	0.762^#^	6 (8.9)	14 (11)	0.646^#^
Prolonged jaundice, *n* (%)	18 (16.2)	17 (13.3)	0.595^#^	9 (13.4)	16 (12.6)	0.870^#^
Congenital malformations, *n* (%)	4 (3.6)	5 (3.9)	0.883^¶^	2 (3.0)	5 (3.9)	0.999^¶^
Family history Maternal history of thyroid disease, *n* (%) Hypothyroidism Graves disease/ATD Papillary thyroid carcinoma Family history of CH, *n* (%)	15 (13.5) 14 (12.6) – 1 (0.9) 7 (6.3)	7 (5.5) 6 (4.7) 1 (0.8) – 5 (3.9)	0.054^#^ 0.495^#^	10 (14.9) 10 (14.9) – – 5 (7.5)	6 (4.7) 5 (3.9) 1 (0.8) – 5 (3.9)	**0.014**^#^ 0.297^#^

*Median (Q1-Q3)

^¶^Fisher’s exact test was used

^#^Chi-square test was used

^&^Mann–Whitney *U* test was used

*TCH* transient congenital hypothyroidism, *PCH* permanent congenital hypothyroidism, *NICU* neonatal intensive care unit, *ATD* antithyroid drug

Maternal history of thyroid disease was significantly higher in the eutopic PCH group

The median initial NBS-TSH level was higher in the PCH group than in the TCH group (*p* < 0.001). The median serum TSH level at the time of diagnosis was higher in the permanent group (*p* = 0.027). There was no difference between the groups in the second NBS-TSH and serum FT4 levels at the time of diagnosis. The serum thyroglobulin level at the time of diagnosis was lower in the PCH group (*p* = 0.006).

There was no difference between PCH and TCH regarding their ages at the diagnosis and at the onset of treatment (*p* = 0.768). However, clinical predictors would not have allowed the identification of TCH patients in the newborn period. Additionally, there was no difference in LT4 starting doses between the groups (*p* = 0.527). Urine iodine levels were measured in 23 cases in the PCH group; 11 had iodine deficiency and 2 had iodine excess. In the TCH group, urinary iodine levels were measured in 13 cases; 4 had iodine deficiency and 5 had iodine excess. Due to limited data availability, maternal thyroid antibodies, urinary iodine levels, and thyroid scintigraphy results were not analyzed. The laboratory findings of cases with PCH and TCH are shown in Table 2.

**Table 2:** Clinical and laboratory features of the cases with PCH and TCH at the time of diagnosis

	PCH (*n* = 111)	TCH (*n* = 128)	*p*-value	Eutopic PCH (*n* = 67)	Eutopic TCH (*n* = 127)	*p*-value
Initial NBS-TSH (µIU/mL)*	32.8 (10.70–8.60)	14.8 (9.30–26.0)	**< 0.001**^**&**^	11.7 (7.5–62.3)	15.3 (9.3–26.0)	0.859^&^
Age at first NBS (days)*	5 (4–8)	5 (4–7)	0.192^&^	5.5 (4–8)	5 (4–7)	0.411^&^
Second NBS-TSH (µIU/mL)*	19.10 (9.42–28.0)	18.53 (9.85–33.5)	0.711^&^	18.29 (8.9–27.9)	19.5 (10.2–33.7)	0.470^&^
Age at second NBS (days)*	14.5 (11–18.75)	14 (12.25–17.20)	0.916^&^	16.0 (12.0–18.5)	14 (12.0–17.0)	0.576^&^
Initial serum TSH (µIU/mL)*	56.62 (21.02–100)	34.2 (20.32–84.12)	**0.027**^**&**^	39.0 (18.2–100)	34.4 (20.4–83.4)	0.620^&^
Initial serum FT4 (ng/dl)*	0.76 (0.39–1.02)	0.73 (0.55–0.98)	0.151^&^	0.82 (0.40–1.12)	0.73 (0.56–0.98)	0.937^&^
Time of the diagnosis (days)*	19 (15–25)	18 (12–25)	0.578^&^	18 (14.5–26.5)	18 (13–25)	0.740^&^
Tiroglobulin (ng/ml)*(*n* = 39)	111 (19.8–455)	347 (300–500)	**0.006**^**&**^	111 (24–500)	347 (300–500)	0.227^&^
Iodine status Iodine deficiency, *n* (%) Normal, *n* (%) Iodine excess, *n* (%)	11 (47.8) 10 (43.5) 2 (8.7)	4 (30.8) 4 (30.8) 5 (38.4)	0.146^¶^	4 (36.4) 7 (63.6) –	4 (30.8) 4 (30.8) 5 (38.5)	0.073^¶^
Thyroid ultrasonography						
Total thyroid volume (ml)*	0.49 (0.06–1.02)	0.77 (0.5–1.02)	**< 0.001**^&^	0.78 (0.52–1.46)	0.77 (0.51–1.0)	0.317^&^
Normal, *n* (%) Hyperplasia, *n* (%) Ectopy, *n* (%) Agenesis, *n* (%) Hypoplasia, *n* (%) Hemiagenesis, *n* (%)	53 (47.7) 14 (12.6) 5 (4.5) 16 (14.5) 18 (16.2) 5 (4.5)	116 (90.6) 11 (8.6) – – – 1 (0.8)	< 0.001^¶^	53 (79.1) 14 (20.9) – – – –	116 (91.3) 11 (8.7) – – – –	0.010^#^
Initial LT4 dose (µg/kg/day)*	10 (7.11–12.72)	10 (6.7–12.5)	0.527^&^	10 (7.0–11.0)	10 (7–13)	0.635^&^
Time of the treatment initiation (days)*	18 (14–25)	18 (12.5–25)	0.768^&^	18 (14.5–26.5)	18 (13–25)	0.740^&^

*Median (Q1–Q3)

^¶^Fisher’s exact test was used

^#^Chi-square test was used

^&^Mann-Whitney *U* test was used

*TCH* transient congenital hypothyroidism, *PCH* permanent congenital hypothyroidism, *NBS* newborn screening, *TSH* thyroid stimulating hormone, *FT4* free thyroxine, *LT4* L-thyroxine

In cases with PCH, initial NBS-TSH and initial serum TSH were significantly higher and total thyroid volume was significantly lower. The serum thyroglobulin level at the time of diagnosis was lower in the PCH group

At the final evaluation, TSH level, FT4 level, and LT4 dose were significantly higher in the permanent group than in the transient group (*p* < 0.001, *p* = 0.005, and *p* < 0.001, respectively). The LT4 withdrawal trial was performed at a median age of 36 (34–38) months. The trial of LT4 withdrawal was successful in 94.1% (*n* = 128) of the cases. Eight cases (5.9%) restarted LT4 treatment at the median of 30 days (30–150) after discontinuation of treatment, with median TSH levels of 10.30 (8.78–13.8) µIU/mL. LT4 doses at the withdrawal were significantly lower in the TCH than in the PCH (*p* < 0.001). However, predictors would not have allowed the identification of TCH patients in the newborn period. The clinical and laboratory features of the cases with PCH and TCH at the withdrawal of LT4 are shown in Table 3.

**Table 3:** Clinical and laboratory features of the cases with PCH and TCH at the withdrawal of LT4

	Permanent CH (*n* = 111)	Transient CH (*n* = 128)	*p*-value	Eutopic Permanent CH (*n* = 63)	Eutopic Transient CH (*n* = 125)	*p*-value
Serum TSH (µIU/mL)*	3.88 (2.50–5.50)	2.41 (1.61 -3.33)	**< 0.001**^&^	3.5 (2.40–5.12)	2.43 (1.6–3.3)	**< .0.001**^&^
Serum FT4 (ng/dl)*	1.32 (1.16–1.47)	1.20 (1.08 -1.38)	**0.005**^&^	1.31 (1.16–1.47)	1.20 (1.09–1.38)	0.021^&^
LT4 dose at the withdrawal of treatment (µg/kg/day)*	2 (1.30–2.50)	1.2 (0.9–1.5)	**< 0.001**^&^	1.80 (1.20–2.2)	1.20 (0.9–1.5)	**< 0.001**^&^
Age at the withdrawal of LT4 (months)*	36 (35.80–37.80)	36 (33.80–38.0)	0.634^&^	36 (35.5–39.5)	36 (33.0–38.0)	0.625^&^

*Median (Q1-Q3)

^&^Mann-Whitney *U* test was used

*TCH* Transient congenital hypothyroidism, *PCH* Permanent congenital hypothyroidism, *TSH* Thyroid stimulating hormone, *FT4* Free thyroxine,* LT4* L-thyroxine

Serum TSH level and LT4 dose at the withdrawal of treatment were significantly higher in cases with PCH

**Fig. 2:**
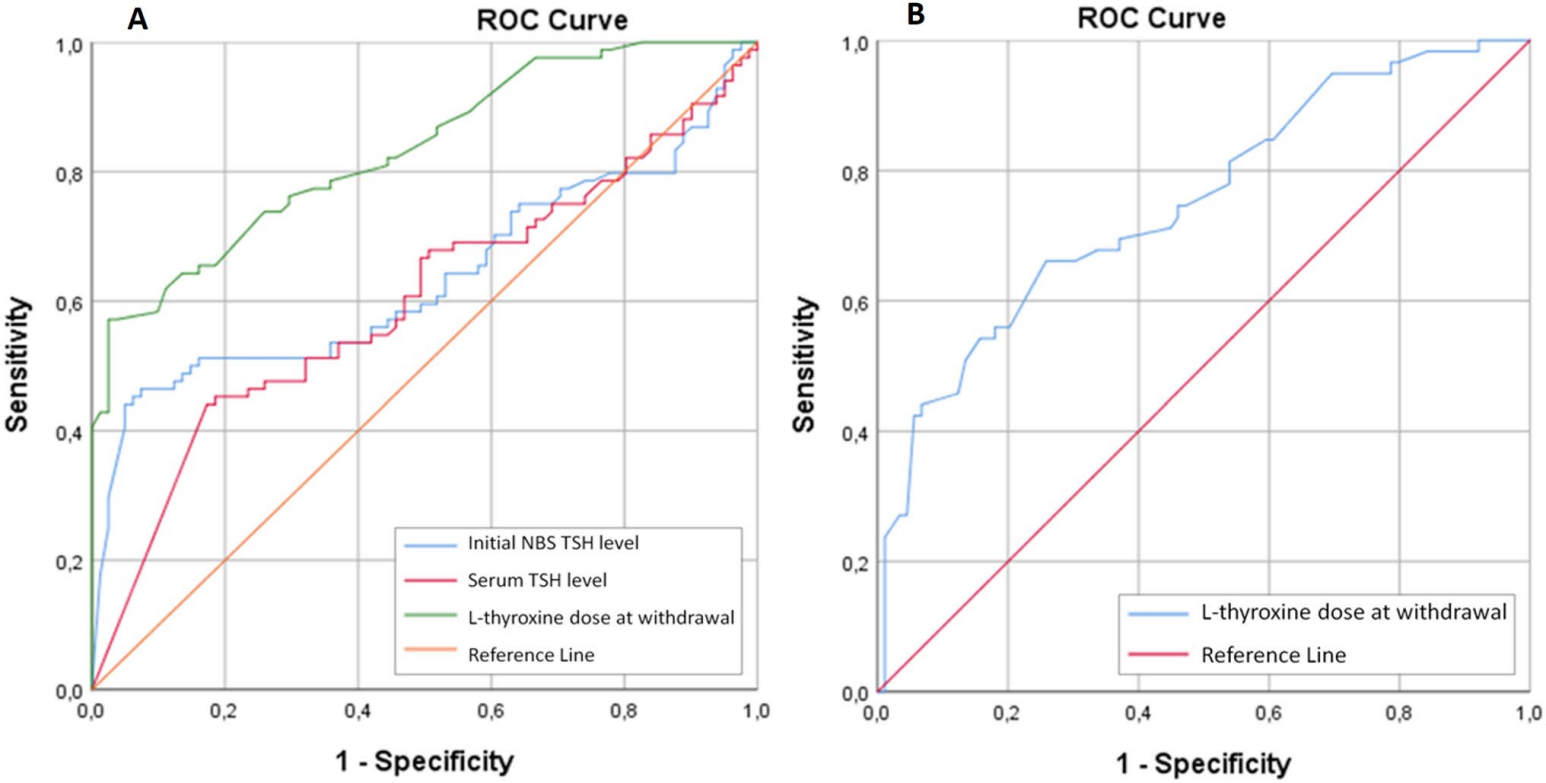
Receiver operating characteristic** (**ROC) curves for initial NBS-TSH level, initial serum TSH level, and LT4 dose at withdrawal, for predicting TCH (**A**). ROC curves for LT4 dose at withdrawal, for predicting eutopic TCH (**B**)

Based on ROC curve analysis to predict TCH, LT4 dose at treatment discontinuation, initial NBS-TSH level, and initial serum TSH level were associated with the diagnosis of TCH. For each parameter, the AUC with 95% CI, the specificity and sensitivity at the optimal cut-off were as follows: initial NBS-TSH level, 0.641 (0.564–0.719), 93.1% and 45.5% at 45.0 μIU/mL; initial serum TSH level, 0.591 (0.515–0.667), 81.5% and 41.3% at 100.0 μIU/mL; LT4 dose on discontinuation of treatment, 0.802 (0.740–0.864), 94.5% and 55.7% at 2.0 µg/kg/day. In patients with LT4 dose < 2.0 µg/kg/day at withdrawal time can predict TCH with a positive predictive value of 91.3% (Fig. 2A).

In eutopic patients, only the LT4 dose at the discontinuation of treatment with a lower sensitivity could predict TCH. LT4 dose < 1.1 µg/kg/day at the time of withdrawal is predictive of TCH with an AUC of 0.750 (sensitivity 84.7%; specificity 40.4%; *p* < 0.001). Whereas, with 66.1% sensitivity and 74.2% specificity, it is < 1.5 µg/kg/day (95% CI, 0.669–0.831; *p* < 0.001) (Fig. 2B).

Multivariate logistic regression analysis showed initial NBS-TSH level [OR: 1.019 (1.003–1.034)] and LT4 dose at the withdrawal [OR: 6.511(3.16–13.41)], were significant predictors of PCH (Table 4).

**Table 4:** Multivariate analysis results for PCH

Variables	Univariate analyses			Multivariate analyses (enter)			Multivariate analyses (backward: LR)		
	OR	95%CI	*p*-value	OR	95%CI	*p*-value	OR	95%CI	p-value
Initial NBS-TSH level	1.031	1.020–1.043	< 0.001	1.021	1.002–1.040	0.029	1.019	1.003–1.034	0.019
Initial serum TSH level	1.009	1.002–1.017	0.017	0.966	0.984–1.009	0.569	–	–	–
LT4 dose at withdrawal	7.243	3.822–13.726	< 0.001	10.269	4.464–23.622	< 0.001	6.511	3.16–13.41	< 0.001

Enter: Nagelkerke R2: 0.476; Hosmer–Lemeshow test *p* value: 0.108; predicted percentage: 78.8%. Backward: LR: nagelkerke R2: 0.416; Hosmer–Lemeshow test *p* value: 0.316; predicted percentage: 78.8%

*PCH* Permanent congenital hypothyroidism, *NBS* Newborn screening, *TSH* Thyroid stimulating hormone, *LT4* L-thyroxine

Initial NBS-TSH level and LT4 dose at the withdrawal were determined as predictors of PCH

## Discussion

Our study is the first multicenter study in Türkiye with long-term follow-up to re-evaluate CH cases referred from NNSP and to determine the frequency of PCH and TCH. We found that 53.6% of 239 patients had TCH. The frequency of TCH has been reported between 11.2 and 88.8% in previous studies [7, 12–14, 20–24, 26–32]. The frequency of TCH in Türkiye was reported as 23.7–73.3% [11, 19, 33–37]. This study showed that 65.5% of CH cases with eutopic thyroid glands had TCH. In every three cases with eutopic thyroid gland, two had TCH. In previous reports, the rate of TCH in patients with eutopic thyroid gland was between 36.4 and 88% [7, 13, 21, 22, 31]. The variations in the frequency of TCH can be attributed to factors such as the methodology of the study, the characteristics of the study population, and the cut-off values for NBS-TSH [11, 19, 30, 32, 33]. One of the most important reasons for the increase in the incidence of CH in recent years is the lowering of the threshold values of NBS-TSH. [2, 10, 38–40]. There are different cut-off values in different countries, and these values have decreased over time; therefore, cut-off values vary over time. The benefit of low cut-off values is controversial [9]. In Türkiye, the TSH cut-off value in NNSP is 5.5 µIU/mL [41]. Iodine deficiency is another reason for TCH. In Türkiye, 74% of pregnant women and 51% of newborns have iodine deficiency [42]. Iodine deficiency or excess was not evaluated in this study due to limited data availability; it is plausible to consider that iodine deficiency may play a significant role in the etiology of TCH within our cohort. High thyroglobulin levels and goiter in the TCH group may result from defects in iodine metabolism [24, 35]. Although serum thyroglobulin levels were measured in a limited number of patients, levels were high in TCH group compared to PCH group which might be related to iodine deficiency. Other reasons for the different frequency are demographic, ethnic, and genetic characteristics, especially when consanguineous societies are taken into account [14, 19, 32, 43, 44]. In addition, some studies have reported that prematurity [24, 38, 45], low birth weight [46, 47], and male gender [33, 35, 48], are risk factors for TCH. In this study, we found no difference between PCH and TCH groups in terms of prematurity and gender.

Thyroid dysgenesis is the most common cause of CH [1]. However, recent reports indicate that the incidence of dyshormonogenesis has increased [21, 22, 24, 38], which is probably due to the decrement of the TSH cut-off limits in NBS programs which enables to diagnosis of mild forms of CH with eutopic gland [24, 38, 40]. In studies conducted in populations with high parental consanguinity, such as Iran, the most common cause of PCH was reported as dyshormononogesis [32, 49–51]. A high consanguinity (55%) rate was associated with an increased risk of autosomal recessive dyshormonogenesis in cases with eutopic PCH [19]. In patients with PCH and eutopic thyroid gland, we hypothesized that dyshormonogenesis could be the underlying etiology. Unfortunately, in this study perchlorate discharge tests or molecular genetic analyses were not performed in all cases for the definite diagnosis of dyshormonogenesis.

Several studies have been conducted to determine the parameters that can distinguish TCH from PCH [7, 13, 27, 29]. Studies have reported conflicting results regarding the use of TSH and FT4 levels at the time of diagnosis as differentiating factors between TCH and PCH [21, 24, 27, 28, 35]. Cut-off values between 28.4 and 31 µIU/mL for initial NBS-TSH [20, 27] and 30.5–97.1 µIU/mL for initial serum TSH have been reported [12, 22, 23, 28]. This study indicates that more than half of the CH cases do not require lifelong treatment, thus markers that enable early detection of TCH are needed. In all cases, initial NBS-TSH > 45 µIU/mL, initial serum TSH > 100 µIU/mL, and the LT4 dose at withdrawal time > 2 µg/kg/day predicted PCH according to ROC analysis.

Further, logistic regression analysis showed that initial NBS-TSH level and LT4 dose at the withdrawal of treatment are the most strong predictors of PCH. In all patients, LT4 dose at withdrawal time < 2 µg/kg/day predicted TCH with 94.5% specificity and 55.7% sensitivity. In the patients with eutopic gland, the only parameter that could predict TCH was LT4 dose < 1.1 µg/kg/day at withdrawal time with an AUC of 0.750 (sensitivity 84.7%; specificity 40.4%). Patients with dysgenesis are expected to have PCH thus differentiation between PCH and TCH is more difficult in patients with eutopic thyroid glands. To differentiate between TCH and PCH based on LT4 doses, various studies have employed different approaches. Some studies have focused exclusively on patients with eutopic thyroid glands, while others have included all patients diagnosed with CH without considering thyroid imaging. In the study of Kang et al. [27], the predictive factors that differentiated TCH vs PCH were NBS-TSH level, and LT4 dose < 4.1 μg/kg/day at age of 2 years in all CH patients. In the study of Fu et al. [28], which all patients with CH were included; LT4 dose > 30 μg/day at day 90 was the best independent predictor of PCH with an accuracy of 93.9%. However, these data may not provide sufficient evidence for re-evaluation as early as 6 months of age. Perhaps, as Zdraveska et al. [23] suggested, in some cases requiring low-dose LT4, earlier re-evaluation may be considered, for example between 2 and 3 years of age. In the same study, initial serum TSH levels and LT4 dose at 3 years of age were important determinants of TCH, while LT4 dose at 1 and 2 years of age were not predictive for TCH in patients with CH [23]. In different studies at 12 and 24 months LT4 doses of < 1.7 µg/kg/day [21],  < 3.25 µg/kg/day [20] and < 1.45 µg/kg/day [21],  < 3.25 µg/kg/day [20], 2.30 µg/kg/day [7], respectively, have been reported to predict TCH in patients with eutopic thyroid gland. When LT4 cut-off doses were evaluated at 6, 12, and 24 months, the AUCs at 6 and 12 months were lower than at 24 months suggesting that early LT4 doses may not accurately predict TCH, and that early LT4 discontinuation can cause irreversible damage [7]. Nevertheless, LT4 dose thresholds for estimating TCH at 6 and 12 months have been reported as 3.2, 2.2 μg/kg/day [13, 22], and 2.5 μg/kg/day [13], respectively, in CH patients with eutopic thyroid glands. In the literature, there are LT4 cut-off doses at different ages suggesting differentiation between TCH and PCH [7, 11, 13, 19]. In this study, we established LT4 cut-off values at the time of withdrawal, which were determined individually for each patient. The timing of withdrawal was determined based on the assessment of thyroid functions and LT4 dosage, rather than a fixed age criterion. Given the ongoing brain development during the first 2 years of life, discontinuation of treatment was not preferably attempted. Levothyroxine dose might suggest the timing for withdrawal.

Withdrawal time is based on the etiologic diagnosis, LT4 dose, and the age of the patient. The safest way seems to be withdrawing treatment after 3 years of age as suggested in the latest AAP guidelines [18]. But if the patient has the tendency to become hyperthyroid despite a low LT4 dose, it is logic to withdraw treatment and follow-up. The recent European Society for Pediatric Endocrinology and the European Society for Endocrinology guidelines addressing stopping treatment after 6 months of age with gland in situ and LT4 dose < 3 μg/kg/day might be unsafe. The practice of conducting an early trial of withdrawal of LT4 treatment raises concerns regarding the potential adverse effects on neurodevelopment in patients who do not respond successfully to the trial and experience a recurrence of hypothyroidism. Thus, it has been reported in the literature that 62–86% of eutopic CH cases with mildly elevated NBS-TSH have PCH [38–40]. However, there is no consensus on TCH predictors and the withdrawal algorithm.

In this study, LT4 withdrawal trial was performed at the median age of 36 months. Levothyroxine treatment withdrawal was considered, in 22.6% between 2 and 3 years of age, in 12.5% younger than 2 years of age, and in 64.9% older than 3 years of age. The age when LT4 treatment is discontinued varies in different reports [11, 13, 35].

The multicenter design of this study which revealed the frequency of TCH in 17 centers in Türkiye is a notable strength. However, there are limitations inherent to its retrospective design, as certain factors such as thyroid scintigraphy, genetic analysis, and environmental influences were not evaluated in all cases. These additional evaluations could have provided further insights into the underlying etiology of TCH.

## Conclusion

In conclusion, we showed that the frequency of TCH was 53.6% in our cohort and 65.5% in CH cases with the eutopic thyroid gland. The LT4 discontinuation success rate was 94.1%. As the most important finding, initial NBS-TSH level and LT4 dose at discontinuation were determined as the predictors of TCH. At withdrawal time, LT4 dose < 2.0 µg/kg/day and initial NBS-TSH level < 45 µIU/mL were the best cut-offs that indicated TCH. In the patients with eutopic gland only LT4 dose at withdrawal time < 1.1 µg/kg/day was predictive of TCH. Nevertheless, the evaluation of genetic and environmental factors at the time of diagnosis, elucidating the underlying etiology may help in determining the cases with TCH and the time of LT4 withdrawal.

## Data Availability

The datasets generated and analyzed during the current study are available from the corresponding author on reasonable request.
